# Human Vav1 Expression in Hematopoietic and Cancer Cell Lines Is Regulated by c-Myb and by CpG Methylation

**DOI:** 10.1371/journal.pone.0029939

**Published:** 2012-01-11

**Authors:** Lena Ilan, Shulamit Katzav

**Affiliations:** Developmental Biology and Cancer Research, Institute for Medical Research Israel-Canada, Hadassah Medical School, Hebrew University, Jerusalem, Israel; Université Paris-Diderot, France

## Abstract

Vav1 is a signal transducer protein that functions as a guanine nucleotide exchange factor for the Rho/Rac GTPases in the hematopoietic system where it is exclusively expressed. Recently, Vav1 was shown to be involved in several human malignancies including neuroblastoma, lung cancer, and pancreatic ductal adenocarcinoma (PDA). Although some factors that affect *vav1* expression are known, neither the physiological nor pathological regulation of *vav*1 expression is completely understood. We demonstrate herein that mutations in putative transcription factor binding sites at the *vav*1 promoter affect its transcription in cells of different histological origin. Among these sites is a consensus site for c-Myb, a hematopoietic-specific transcription factor that is also found in Vav1-expressing lung cancer cell lines. Depletion of c-Myb using siRNA led to a dramatic reduction in *vav*1 expression in these cells. Consistent with this, co-transfection of c-Myb activated transcription of a *vav1* promoter-luciferase reporter gene construct in lung cancer cells devoid of Vav1 expression. Together, these results indicate that c-Myb is involved in *vav*1 expression in lung cancer cells. We also explored the methylation status of the *vav*1 promoter. Bisulfite sequencing revealed that the *vav*1 promoter was completely unmethylated in human lymphocytes, but methylated to various degrees in tissues that do not normally express *vav*1. The *vav*1 promoter does not contain CpG islands in proximity to the transcription start site; however, we demonstrated that methylation of a CpG dinucleotide at a consensus Sp1 binding site in the *vav*1 promoter interferes with protein binding *in vitro*. Our data identify two regulatory mechanisms for *vav*1 expression: binding of c-Myb and CpG methylation of 5′ regulatory sequences. Mutation of other putative transcription factor binding sites suggests that additional factors regulate *vav1* expression as well.

## Introduction

The specification and maintenance of tissues is a fundamental aspect of development, mediated in part by hierarchical networks of transcription factors and *cis*-regulatory elements that control gene expression. Additionally, somatic epigenetic inheritance, particularly through DNA methylation and chromatin remodeling, plays a critical role in regulating the development of multicellular eukaryotic organisms [1].

Hematopoiesis is one of the best-studied examples of development and differentiation from stem cell maintenance to lineage commitment and differentiation [2]. *Vav*1 expression, which is strictly confined to the hematopoietic system [3], is upregulated in the aorta-gonad-mesonephros (AGM) region of the embryo during the switch from primitive to definitive hematopoiesis [4] and is subsequently expressed only in cells of the adult hematopoietic system [3]. The AGM is an important intraembryonic source of hematopoietic stem cells and the appearance of these stem cells correlates with the upregulation of *vav*1 expression. Definitive hematopoietic stem cells appear to differentiate from the ventral hemogenic endothelium of the dorsal aorta and enter the developing circulatory system to seed the fetal liver [5], where erythrocytic, myeloid, and lymphoid lineages develop. In newborn and adult mice, *vav*1 is expressed specifically in hematopoietic cells from the thymus, lymph node, bone marrow, and spleen [5]. Vav1 was first identified in a screen for oncogenes in which NIH3T3 cells were transfected with DNA from several esophageal carcinomas [3]. Nucleotide sequence analysis revealed that the Vav1 oncogene was activated *in vitro* and the isolated mutant form was not present in the original tumor sample [3].

Several characteristic structural motifs enable Vav1's signal transducer function [6]–[8]. The best-known function of Vav1 is as a GDP/GTP exchange factor for Rho/Rac, a function strictly controlled by tyrosine phosphorylation [6]–[8]. Rho/Rac activation leads to cytoskeletal rearrangement during activation of T cells [6]–[8]. There is also increasing evidence suggesting that Vav1 has other effects that are independent of its exchange activities, including modulating the JNK, ERK, Ras, NF-kB, and NFAT pathways. These effects are likely mediated by Vav1's modular domains via interaction with other proteins, including Shc, NCK, SLP-76, GRB2, and Crk [6]–[8].

We initially characterized the *vav*1 promoter 20 years ago [9]. Analysis of the promoter region determined the transcription start sites and indicated that the promoter lacks identifiable core promoter elements such as a TATA box or an initiator. However, it does contain several consensus binding sites for both ubiquitous (e.g., Sp1, AP-1, and AP-2) and tissue-restricted (GATA, Myb, OCT, and ETS proteins) transcription factors [9]. The murine promoter was cloned subsequently [10], [11]. RNase protection experiments were performed on mRNA from cell lines representative of diverse hematopoietic lineages. All these RNA samples yielded a pattern of fragments corresponding to a cluster of major and minor start sites 95 to 133 bp upstream of the translation initiation codon, near the multiple start sites mapped for the human *vav*1 mRNA [10], [11]. Thus, a single *vav*1 promoter appears to be operative throughout the hematopoietic compartment. As expected, the promoter of *vav*1 was shown to drive transgene expression in multipotent hematopoietic stem cells residing in the bone marrow of adult mice as well as in various hematopoietic organs [12]–[14]. For instance, several independent lines of human NPM-ALK transgenic mice were generated by using the hematopoietic cell-specific *vav*1 promoter. This new transgenic model provided a system for investigating the oncogenic events mediated by NPM-ALK in situ [14]. Also, lentiviral vectors expressing the common cytokine receptor gamma chain under the control of the proximal *vav*1 gene promoter were shown to be effective for correction of signaling defects and the X-linked severe combined immunodeficiency (SCID-X1) disease phenotype in a murine model [13].

Although *vav*1 promoter has been used to drive specific expression in the hematopoietic system, little is known about the transcription factors that regulate its activity. In a series of studies, Denkinger *et al.* demonstrated that PU.1 is essential for transcriptional activity of the *vav*1 promoter in myeloid cells, but not in other hematopoietic cells [15]. Moreover, Vav1 and PU.1 are recruited to the CD11b promoter in APL-derived promyelocytes, suggesting that the ATRA-induced increase of Vav1 expression and tyrosine phosphorylation may be involved in recruiting PU.1 to its consensus sequence on the CD11b promoter and, ultimately, in regulating CD11b expression during the late stages of neutrophil differentiation of APL-derived promyelocytes [16].

Vav1 mutations have not been detected so far in human cancer. Thus, although truncated versions of Vav1 lacking the amino terminus transform NIH3T3 fibroblasts [9], [17] and synergize with active Ras in transformation [18], [19], their role in human tumorigenesis is disputed [20]. A number of groups, including ours, have detected the ectopic expression of Vav1 in neuroblastoma [21], pancreatic ductal adenocarcinomas (PDA) [22] and lung cancer [23]. These findings suggest that ectopic Vav1 expression may be a more general phenomenon affecting additional tumor types. Determining what drives aberrant Vav1 expression in tissues outside the hematopoietic system is important for understanding Vav1's involvement in human cancer.

Our current study reveals the involvement of the hematopoietic transcription factor c-Myb in the expression of *vav*1 in lung cancer cells. We also demonstrate the contribution of CpG dinucleotide methylation of the *vav*1 promoter to its expression in hematopoietic and cancer cells.

## Materials and Methods

### Cell lines

Jurkat (acute T cell leukemia, kindly given to us by Dr. Weiss [24]), U937 (monocytes, histiocytic lymphoma [25]), H441 (lung papillary adenocarcinoma, kindly given to us by Drs. Gazdar and Minna [26]), H460 (large cell lung cancer kindly given to us by Drs. Gazdar and Minna [26]) and H358 (bronchioalveolar Non-Small Lung Carcinoma, kindly given to us by Drs. Gazdar and Minna [26]) cells were grown in RPMI medium. Panc1 (pancreatic duct epithelioid carcinoma, kindly given to us by Dr. Billadeau [22]) and A549 (lung epithelial carcinoma, kindly given to us by Drs. Gazdar and Minna [26]) cells were grown in DMEM medium (Sigma). All media was supplemented with 10% Fetal Bovine Serum (FBS), Penicillin-Streptomycin and L-Glutamine (Biological Industries, Israel) and cells were maintained at 37°C with 5% CO_2_.

### Promoter-reporter constructs and site-directed mutagenesis

The firefly luciferase vector pGL3-basic and *Renilla* luciferase vector pRL-CMV (Promega, USA) were used in this study. The proximal 5′ region of human *vav*1 gene [−287 to +301 relative to the transcription start site (TSS)] was cloned with primers lil30 and lil32 (Table 1) and inserted in-frame into pGL3-basic reporter vector using SacI and XhoI restriction sites to create construct Le2. Le2 was then used as the template to generate a series of point mutations and deletions (Table 1). The PCR reactions were performed using Pfu-X Polymerase (Jena Bioscience, Germany) under the following conditions: 94°C, 5 min; 35 cycles of (94°C for 15 seconds, 55–62°C for 30 seconds, 72°C for 1 min for lil30 and lil31, and for 4 min for the other primer pairs as described in Table 1). The PCR products were purified from 1% agarose gel using the Wizard SV Gel and PCR Clean-Up System (Promega, USA). The lil30-32 fragment was digested with SacI and XhoI restriction enzymes and ligated into pGL3 vector using Fast-Link DNA ligation kit (Epicentre, USA). PCR products of site-direction mutagenesis were self-ligated.

**Table 1:** Primers used for preparation of the *vav*1 promoter constructs*.

Construct	Primer name	Sequence (5′->3′)
Le2 (Sense)	lil30	AAGAGCTCGAAGTGGGTGAATTCTGGG
Le2 (Antisense)	lil32	AACTCGAGCTGGGACATCTGGGGC
Le7 (Sense)	lil40	CAGGCAAAGAAGAGGAAG
Le7 (Antisense)	lil41	TTTCTGTCGCCCTGAGAG
Le12 (Sense)	lil38	CAGGCAAAGAAGAGGAAG
Le12 (Antisense)	lil39	TAACTGGTGCCCTGAGAGG
Le13 (Sense)	lil59	GAAAAAGTGGTAGCACTAGCTGTC
Le13 (Antisense)	lil60	TGAGAGGGGGTGGAGGA
Le15 (Sense)	lil69	GAAAAAGTGGTAGCACTAGCTGTC
Le15 (Antisense)	lil70	TTCTTTGCCTGTAACTGTCG
Le17 (Sense)	lil57	GTAGCACTAGCTGTCGC
Le17 (Antisense)	lil58	CTGTAACTGTCGCCCTGA
Le19 (Sense)	lil71	GCAAAGAAGAGGAAGTGGT
Le19 (Antisense)	lil72	CTGTAACAATCGCCCTGAG
Le20 (Sense)	lil75	GAAAGAGATGTCAGATTCTG
Le20 (Antisense)	lil76	CTCGACACGGCCTG

*The underlined sequences correspond to the nucleotide replacement mutations.

### Transient transfections and luciferase reporter assay

Cells were seeded and transfected after 24 h under conditions shown in Table 2. Cells were harvested 24 or 48 hrs after transfection. Luciferase reporter assays were performed with Dual-Luciferase Reporter System (Promega, USA) using Luminometer Mithras (Berthold Technologies, Germany). For the c-Myb overexpression experiments, 1 µg of c-Myb expressing plasmid (Open biosystems, USA #6069320) was co-transfected with Le2 reporter construct and *Renilla* into H460 cells. The cells were harvested 24 hrs after transfection. Methylated Le2 plasmid was prepared using CpG methyltransferase (M.SssI) (New England Biolabs, USA).

**Table 2:** Transfection conditions for different cell lines used in this study.

Cell line	Plate	Density	Total volume (ml)	Transfection reagent	Le2 (µg)	*Renilla* (ng)
Jurkat	12 well	5×10^5^/ml	4	Electroporation (BioRad, USA)	2	20
U937	24 well	5×10^5^/ml	1	Lipofectamine 2000 (Invitrogen,USA)	2	20
H441	6 well	5×10^5^/well	4	JetPEI (Polyplus, France)	2	20
H460	6 well	2×10^5^/well	4	JetPEI (Polyplus, France)	2	20
Panc1	6 well	10^5^/well	4	JetPEI (Polyplus, France)	2	20
A549	6 well	10^5^/well	4	JetPEI (Polyplus, France)	2	20

### Bisulfite sequencing

DNA from normal human tissues was obtained from BioChain (USA). Bisulfite reaction was performed using EZ DNA Methylation-Direct Kit (Zymo Research, USA). The sequences of interest were amplified by PCR with primers lil11 (ACACACCTAAACCCCATC) and lil53 (GGGTTGGATTAGATAGAGGA) using 2 µl of 10 µl total volume of the bisulfitization reaction, Tm = 55°C, 35 cycles. PCR products were purified and cloned into the pGEMT plasmid (Promega, USA). Ligated plasmids were used to transform DH5α competent cells. PCR was then performed on bacterial colonies with standard primers for T7 and SP6 promoters. The PCR products of correct length were sequenced by Macrogen (Korea).

### Electrophoretic mobility shift assay (EMSA)

Nuclear extracts were isolated as described [15]. To obtain short double stranded DNA probes, single-stranded oligonucleotides (IDT, USA) (Table 3) were annealed and then labeled with Digoxigenin Oligonucleotide 3′-End Labeling Kit (Roche, Switzerland). The long wild type Le2 probe was created by PCR with Digoxigenin labeled primers lil46 (5′-GCTGCAGGTGCTCC-3′) and lil47 (5′-CCTGCTCGCCTGTG-3′) using the Le2 plasmid as a DNA template. For probes that containing mutations, same primers were used and corresponding mutated plasmid was used as a template. The DNA-protein binding reactions were performed at room temperature for 15 min in a total volume of 20 µl. The reaction contained 60 fmole labeled DNA probe, 4 µg nuclear extract, 2 µg poly(dI•dC) and binding buffer (1 mM Tris pH 7.5, 7.5 mM NaCl, 1 mM EDTA, 0.1 mM DTT, 0.7% glycerol). For competition assays, 1- to 10-fold unlabeled double-stranded DNA was added in the reaction mix 10 min prior to the labeled probe addition. Reaction mixtures were then separated on 4% non-denaturing polyacrylamide gel. Electrophoresis was performed in 0.5×TBE buffer at room temperature at 60 volt for 1 hr. The DNA-protein complexes were transferred to positively charged nylon membrane (Roche, Switzerland), cross-linked by UV using UV Stratalinker 2400 (Stratagene, USA). Digoxigenin-labeled DNA was detected with DIG Gel Shift Kit, 2^nd^ generation, using CDP-Star substrate (Roche, Switzerland). Images were exposed to X-ray films for 15–20 min.

**Table 3:** Oligonucleotides used in EMSA, introduced mutations are underlined.

Description	Oligonucleotides	Sense (5′->3′)	Antisense (5′->3′)
E2F/NF-e/c-Myb and TCF μ/PU.1/ELF1 binding sites (−45 to 0)	lil157-158	CCCTCTCAGGGCGACATTACAGGCAAAGAAGAGGAAGTGGTAGC	GCTACCACTTCCTCTTCTTTGCCTGTAACTGTCGCCCTGAGAGGG
−27–28 TT>AA substitution (−45 to 0)	lil159-160	CCCTCTCAGGGCGACAGAAACAGGCAAAGAAGAGGAAGTGGTAGC	GCTACCACTTCCTCTTCTTTGCCTGTTTCTGTCGCCCTGAGAGGG
−32–33 GA>AC substitution (−45 to 0)	lil161-162	CCCTCTCAGGGCACCAGTTACAGGCAAAGAAGAGGAAGTGGTAGC	GCTACCACTTCCTCTTCTTTGCCTGTAACTGGTGCCCTGAGAGGG
E2F/NF-e/c-Myb binding site (−39 to −22)	lil87-88	CAGGGCGACAGTTACAGG	CCTGTAACTGTCGCCCTG
−27–28 TT>AA substitution (−39 to −22)	lil89-90	CAGGGCGACAGAAACAGG	CCTGTTTCTGTCGCCCTG
−32–33 GA>AC substitution (−39 to −22)	lil147-148	CAGGGCACCAGTTACAGG	CCTGTAACTGGTGCCCTG
Sp1 binding site (−160 to −141)	lil79- 80	GTGTCGAGTGGGCGGAAGAA	TTCTTCCGCCCACTCGACAC
CpG 3+4 methylated (−160 to −141)	lil85-86	GTGTmetCGAGTGGG^met^CGGAAGAA	TTCTT^met^CCGCCCACT^met^CGACAC
CpG3 methylated (−160 to −141)	lil81-84	GTGT^met^CGAGTGGGCGGAAGAA	TTCTTCCGCCCACT^met^CGACAC
CpG4 methylated (−160 to −141)	lil82-83	GTGTCGAGTGGG^met^CGGAAGAA	TTCTTC^met^CGCCCACTCGACAC

### Annealing

Lyophilized complementary oligonucleotides were diluted to 100 µM, and then mixed in equimolar concentrations in annealing buffer (10 mM Tris, pH 7.5–8.0, 50 mM NaCl, 1 mM EDTA) for final concentration of 3 µM each. Annealing mixture was heated to 100°C for 5 min and slowly cooled to 30°C during 1 hr.

### RNA isolation and reverse transcription

RNA isolated with TRIzol reagent (Invitrogen, USA). Total RNA (2 µg) was reverse-transcribed with M-MLV polymerase and random hexamer primer (Promega, USA) in a total reaction volume of 20 µl. PCR was performed with GoTaq Green Master Mix (Promega, USA) and 1 µl of the cDNA; for actin detection cDNA was diluted tenfold. Primers for the different genes are listed in Table 4.

**Table 4:** Primers that were used for gene expression analysis.

Gene	Primer name	Sequence (5′->3′)
*vav*1	lil 7	CACAGGCGAGCAGGG
*vav*1	lil 8	CACAGAAGGACACCATCC
c-*myb*	lil 67	TCAGGAAACTTCTTCTGCTCACA
c-*myb*	lil 68	AGGTTCCCAGGTACTGCT
*actin*	lil 14	ACCCTACTCACCTATAAAAC
*actin*	lil 15	CGCAGCTCATTGTAGAAG

### Immunoblot Assay

Jurkat, H441 and H460 cell lines were processed for protein extraction and Western blotting using standard procedures. Briefly, the cells were washed twice in PBS and lysed in lysis buffer (50 mM Tris pH 7.6, 150 mM NaCl 5 mM EDTA, 0.5% NP40) containing protease inhibitors (0.1 mM phenyl-methyl sulphonyl fluoride; Halt Protease Inhibitor cocktail (Thermo Scientific), 5 mM EDTA), kept at 4°C for 15 min, centrifuged for 10 min at 12000 g and supernatants were collected. Twenty five µg of protein lysates was resolved in 8% SDS-PAGE. Resolved proteins were transferred on to the nitrocellulose membrane. After quick washing in TBST (50 mM Tris.HCl, pH 7.4, 150 mM NaCl, 0.2% Tween), the membranes were blocked in 3% BSA for 1 hr and then incubated with primary antibodies for c-Myb (Santa Cruz),Vav1 (Upstate Biotechnology Inc, USA), and β-actin (Santa Cruz) (diluted in 1% BSA in TBST) overnight at 4°C. The membrane was then washed (3×10 min) in TBST at room temperature and probed with 1∶10000 diluted horseradish peroxidase-conjugated anti-mouse or anti-rabbit secondary antibodies for 1 hr at room temperature and washed 3×10 min with TBST. The signal was detected with an ECL chemiluminescence kit (Pierce, USA).

## Results

### The minimal promoter region of human vav1 and its tissue specific expression

**Figure 1:**
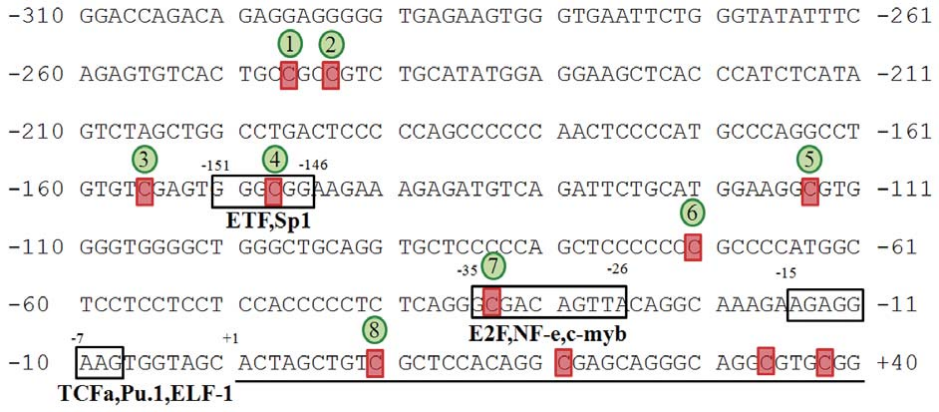
Nucleotide sequence of the 5′ minimal regulatory region of the human *vav*1 gene. Boxes indicate putative binding sites for various transcription factors as predicted by bioinformatics. Their location is indicated relative to the transcription start site (+1 position). Putative sites for CpG methylation are highlighted in red, their arbitrary serial numbers are circled in green.

The sequences of the minimal promoter region of human and murine *vav*1 have been published (Gene ID: 7409 and Gene ID: 22324, respectively). Analysis of the human *vav*1 promoter with TESS (Transcription Element Search System; http://www.cbil.upenn.edu/cgi-bin/tess/tess) reveals numerous putative binding sites for transcription factors including ETF, Sp1, E2F, NF-e, c-Myb, TCFα, PU.1 and ELF-1 (Figure 1, boxed). In addition, the promoter contains 8 potential CpG methylation sites (Figure 1, highlighted in red and numbered arbitrarily 1–8).

**Figure 2:**
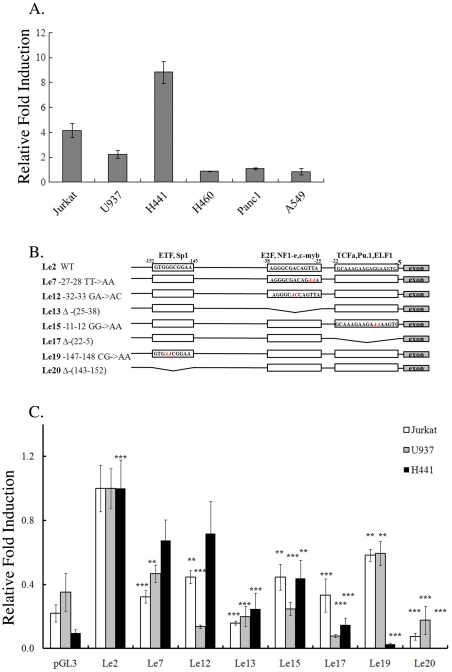
The *vav*1 5′ untranscribed sequences contain cell-type specific cis-regulatory elements. (A) Expression of wild-type (wt) luciferase reporter gene (Le2) in cell lines from various tissue origins. Le2 was transfected into the cell lines as described in Materials and Methods and luciferase activity was measured 24 hr later. Data show luciferase activity normalized to *Renilla* transfection efficiency control and calculated relative to the luciferase activity of an empty vector expression, pGL3. The experiments were repeated five times. (B) Schematic map of the 5′ regulatory region of the human *vav*1 gene. Three putative transcription factor binding sites are highlighted by boxes. The changes introduced in these regions are as follows: nucleotide substitutions (red) and deletions (crooked lines). (C) The effect of these mutations/deletions was analyzed in Jurkat T cells, U937 myeloid cells and H441 lung cancer cells. Following transfection with plasmids containing luciferase under wt (Le2) or mutated *vav*1 promoter, the luciferase activity was measured and fold induction of activity was calculated relative to the activity of Le2. Experiments were repeated five times. Statistics were performed using the unpaired student T test. (**) indicates p<0.05 value and (***) indicates p<0.01.

Tissue-specific expression of genes can be achieved by activity of tissue-specific transcription factors as well as by regulation of the affinity between DNA-binding factors and promoter sequences. To identify regulatory sequences required for the restricted expression of *vav*1, we generated a pGL3-*vav*1 reporter construct (Le2) containing the minimal regulatory sequences of *vav*1 proximal promoter region [from nucleotide (nt) −287 to +301 relative to Transcription Start Site (TSS)] upstream of a luciferase reporter gene. To validate that the expression of Le2 corresponds with the endogenous expression of *vav*1 in cells of different histological origins, the plasmid was transfected into Jurkat T cells and U937 monocyte cells in which *vav*1 is expressed physiologically, and into H441 lung cancer cells, where it is aberrantly over-expressed [23]. Le2 was also transfected into *vav*1-negative cell lines: lung cancer cells H460 and A549 [23]) and pancreatic cancer cell line Panc1 [22] (Fig. 2A). Following transfection, luciferase was expressed at high levels in the Vav1-expressing cells (Jurkat, U937 and H441), but its expression level was very low in the *vav*1-negative cell lines (H460, A549 and Panc1) Luciferase expression in H441 lung cancer cells was even higher than in Jurkat T cells (Fig. 2A).

To characterize the promoter regions involved in *vav*1 expression, we created several point mutations and deletions in the predicted transcription factor binding sites (indicated in Figure 2B) and tested the expression of reporter constructs bearing these mutations in various cell lines (Figure 2C). Our results clearly show that each nucleotide substitution or deletion in putative transcription factor binding sequences reduced the activity of the promoter compared to the wild type construct, Le2 (Fig. 2C). For some mutants, we also observed significant differences between their expression in Jurkat, U937 and H441 cells. For instance, Le12, Le15 and Le17 are better expressed in Jurkat T cells than in U937 cells, indicating that even among cells of hematopoietic origin, there are differences in the regulation of *vav*1 expression. Le15 and Le17 carry mutations in the PU.1 binding site, supporting the need for PU.1 binding in U937 cells. This is consistent with previous reports of differential requirements for PU.1 for Vav1 expression in different hematopoietic cells [15]. Le7 and Le12, which have base pair substitutions in a putative E2F/NF-e/c-Myb binding site, exhibit significantly reduced luciferase expression in hematopoietic cells; however, these mutations have only a minor effect on luciferase expression in H441 lung cancer cells. Deletion of the entire E2F/NF-e/c-Myb site (Le13) abolishes luciferase expression in all cell lines used in this study. A point mutation in the ETF/Sp1 binding site (Le19) has a smaller effect on reporter gene expression in the hematopoietic cell lines than in the lung cancer cell line, but again, deletion of the entire binding site (Le20) abolishes luciferase expression in all cell lines examined in this study. Mutagenesis in the TCFα/PU.1/ELF-1 binding site (Le15 and Le17) abates luciferase expression in a similar manner in all cell lines. Thus, our results point to the involvement of several transcription factors in regulating Vav1 expression in cells of different histological origin.

**Figure 3:**
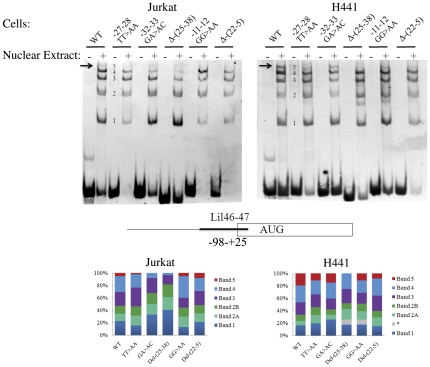
Mutations at various transcription factors binding sites affect protein complexes formation at the *vav1* promoter. Electrophoretic mobility shift assay (EMSA) with Jurkat and H441 nuclear extracts was performed in the presence of lil46-47 digoxigenin-labeled probe (nucleotides −98 to +28 of *vav1* promoter). To produce the mutant oligonucleotides, the corresponding mutated plasmids (shown in Fig. 2B schematic) were used as template for the PCR. A schematic of *vav*1 5′ regulatory sequences, exon 1 and relative oligonucleotide position is shown at the bottom. Bound protein complexes are numbered 1 to 5. The arrow shows the position of complex 5, the heaviest complex that is sensitive to the mutations introduced into the oligonucleotide sequence. The bottom panels of the figure schematically show the relative intensity of bands 1–5 of the EMSA experiment as determined by densitometry (ImageJ software).

To determine if these mutations alter binding of nuclear proteins to the *vav*1 promoter, we performed an electrophoretic mobility shift assay (EMSA). Digoxigenin-labeled double-stranded oligonucleotides encompassing nucleotides −98 to +25 (lil 46-47) of the *vav*1 promoter (Fig. 3) were used as probes in the presence of nuclear extracts from Jurkat and H441 cells. Wild type oligonucleotide and oligonucleotides with mutations corresponding to the mutations in the reporter constructs (Fig. 2B) were used. The protein complexes that assemble on the wild type DNA sequence in the nuclear extract appear as five major bands (labeled 1–5) in both cell lines; however, the intensity of these bands differs between the two (Fig. 3 bottom). Thus, band 5 exhibits a higher intensity in nuclear extract from H441 cells than in nuclear extract from Jurkat T cells (19.2% vs. 4.9%). Binding of the protein complex represented by band 5 was partially or completely lost in some of the mutated sequences used in this study (Fig. 3). This band completely disappeared in the GA>AC and deletion (−25–38) mutants in Jurkat T cells, while in H441 it disappeared only in the −25–38 deletion mutant, thus potentially corresponding to the loss of promoter activity of the deletion mutation in H441 cells (Fig. 2C). Additionally, the intensity of band 4 is lower in the GA>AC and deletion (−25–38) mutants in Jurkat T cells, while it does not change in H441 cells (Fig. 3). These results clearly indicate the there are differences in protein complexes assembled on the promoter region in cells from different origins. Our data indicates that the region of the *vav*1 promoter between −98 and +25 is critical for *vav*1 expression in various cell lines and encodes putative binding sites for several transcription factors.

### c-Myb is involved in regulation of vav1 expression in hematopoietic and lung cancer cells

**Figure 4:**
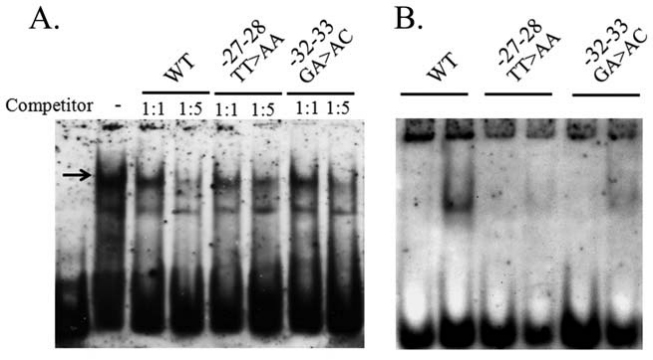
Mutations at the E2F/NF-e/c-Myb binding site affect binding of protein complexes to the *vav1* promoter *in vitro*. (A) Electrophoretic mobility shift assay (EMSA) with Jurkat nuclear extracts was performed in the presence of digoxigenin-labeled probe spanning nucleotides −45 to 0 of *vav1* promoter and containing E2F/NF-e/c-Myb and TCFα/PU.1/ELF1 binding sites (lil157-158; Table 3). The competition assay was performed with the labeled oligonucleotide and unlabeled competitor oligonucleotides with point mutations as indicated in Table 3 in molar ratio of 1∶1 and 1∶5. The arrow shows the position of the complex that demonstrates sensitivity to the introduced mutations. (B) EMSA performed with labeled oligonucleotide containing only E2F/NF-e/c-Myb binding site (lil 87-88; Table 3).

While PU.1 exhibits specificity for the myeloid cell lineage, as reported previously [27]–[29], most of the other transcription factors seem to be ubiquitously expressed, albeit at different levels. One transcription factor that might affect the level of *vav*1 expression in lung cancer cells is c-Myb. c-Myb is highly expressed in immature hematopoietic cells and is down-regulated during differentiation [30], [31]. To determine whether the c-Myb binding site in *vav*1 promoter participates in generation of protein complexes, we used a double-stranded oligonucleotide encompassing the binding sites for the transcription factors E2F/NF1-e/c-Myb and TCFα/PU.1/ELF1 (lil 157-158, Table 3). Mutations introduced in the c-Myb binding site (TT>AA) affected the affinity of Jurkat T cells protein complex as determined by a competition assay (Fig. 4A), while the effect of a mutation in the E2F binding site (GA>AC) had a lesser effect (Fig. 4A). By using a shorter oligonucleotide that contains only the c-Myb/E2F binding site (Table 3, lil87-88); we noticed that only one protein complex is formed with nuclear extracts of Jurkat T cells (Fig. 4B). This protein complex is totally disrupted when the TT>AA mutation (c-Myb binding site) is used, while the GA>AC mutation (E2F binding site) still forms a similar band to the wild-type oligonucleotide (WT), albeit at a lower level (Fig. 4B). In agreement with the results of Figure 4A, mutation in the c-Myb impair the ability of the protein complex to bind DNA and GA>AC substitution has a lesser but significant effect.

**Figure 5:**
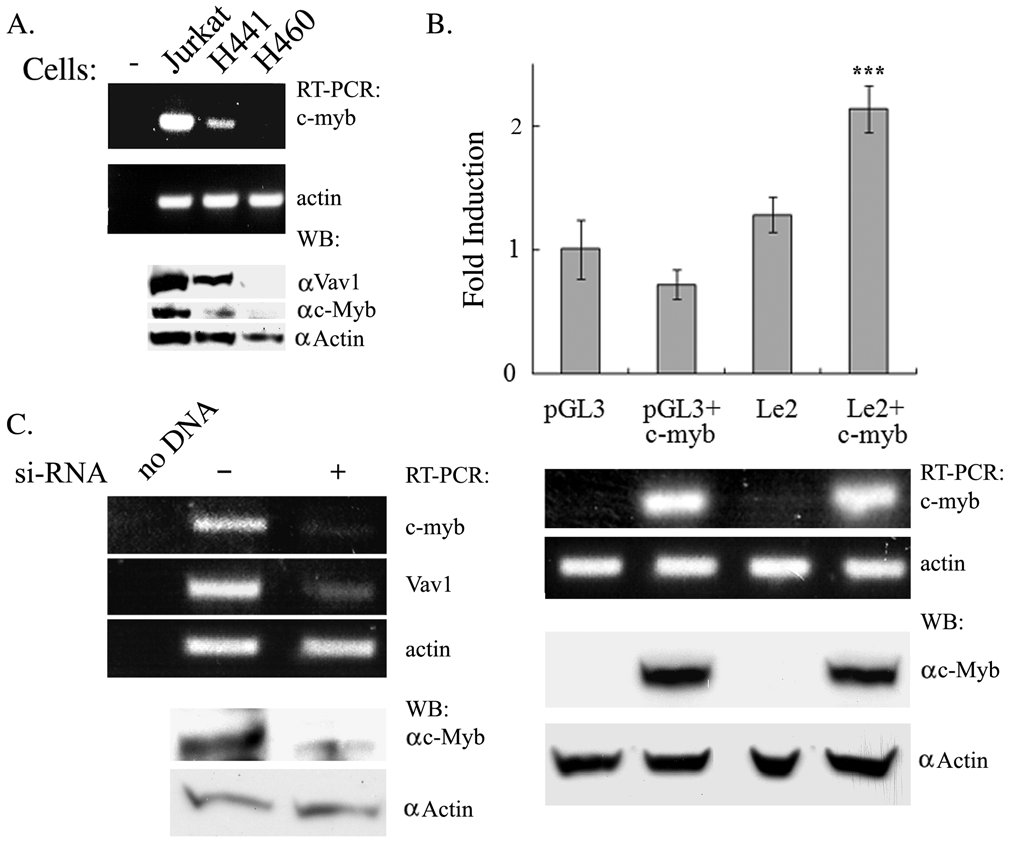
C-Myb is involved in regulation of *vav*1 expression in lung cancer cells. (A) Endogenous expression of *c-myb* mRNA in Jurkat T cells, H441 (*vav*1-positive) and H460 (*vav*1-negative) lung cancer cell lines was detected by RT-PCR and western blotting. (B) Empty vector pGL3 or the Le2 wt reporter construct was transfected either alone or with a c-Myb-expressing plasmid into H460 lung cancer cells (as in Materials and Methods). Luciferase activity was measured 24 hr after transfection (top panel). Luciferase activity is expressed as fold induction relative to basic pGL3 expression. Values are the mean of five independent experiments; significance was determined using the unpaired student T test. (***) indicates p<0.01. The bottom panel shows the level of *c-myb* and actin mRNA and protein expression in the transfected cells as determined by RT-PCR and Western blotting respectively. (C) H441 lung cancer cells were transfected with either scrambled DNA (-) or with siRNA against c-Myb. Seventy-two hours later, the mRNA levels of *c-myb*, *vav1* and *actin* were detected by RT-PCR.

To further determine whether c-*myb* is involved in Vav1 expression, we analyzed its expression in cells of different histological origins and found that *c-myb* mRNA and protein is present in Jurkat T cells and at lower levels in H441 lung cancer cells, but is hardly detectable in H460 lung cancer cells that do not express *vav*1 (Fig. 5A). To examine whether c-Myb participates in the regulation of *vav*1 expression, we co-transfected a c-Myb expression vector with either an empty vector or with Le2 into H460 cells (Fig. 5B). Co-trasnfection of *c-myb* with Le2 significantly increases the expression of the reporter gene compared to the expression of Le2 alone (upper panel). We also determine the level of *c-myb* mRNA and protein expression in the transfected cells (lower panel). Down-regulation of *c-myb* by transfection of siRNA into H441 lung cancer cells significantly decreased *vav*1 expression (Fig. 5C). Collectively, these results suggest that c-Myb plays a role in the regulation of *vav*1 expression in epithelial lung cancer cells.

### Methylation of individual CpG sites in human vav1 promoter is important for the regulation of its expression

Changes in DNA accessibility for DNA-binding factors also participate in regulating gene expression. One mechanism that affects DNA accessibility is methylation of CpG dinucleotides at specific protein binding sites [32]. It has been demonstrated that epigenetic modifications, including methylation, play an important role in aberrant *vav*1 expression in pancreatic cancer cell lines [22]. However, this study did not decipher the mechanism in-depth. To begin to assess the role of methylation in regulation of Vav1 expression, we analyzed methylation of the *vav*1 promoter in samples from different normal human tissues (Table 5). About 600 bp of *vav*1 promoter sequences upstream and downstream of the TSS were analyzed by bisulfite sequencing. Strikingly, in lymphocytes, we observed no methylation of any of the putative CpG methylation sites sequenced. In contrast, in DNA from tissues that do not normally express Vav1, we detected various degrees of methylation at sites in the *vav*1 promoter (Table 5). For instance, the methylation level in the pancreas is 48–100%, in the lung the level is between 22–50%, whereas in colon the percentage of methylation is very low (between 4 to 15 percent). These results imply that methylation plays an important role in the regulation of *vav*1 expression.

**Table 5:** Methylation status of CpG dinucleotides in *vav1* promoter in tissues of different histological origin*.

Position	Colon	Pancreas	Stomach	Lymph	Liver	Muscle	Lung	Brain
−247	15	83	41	0	46	33	40	63
−244	7	96	42	0	31	20	40	63
−156	12	74	19	0	31	13	50	75
−148	8	100	59	0	38	33	50	94
−114	4	83	30	0	15	13	50	57
−71	4	48	24	0	18	21	22	54
−34	4	57	21	0	18	7	30	46
+10	8	77	24	0	27	7	33	71
+21	4	82	13	0	0	0	44	71
+34	4	82	39	0	27	14	38	86
+38	15	82	27	0	45	21	38	93
N	27	24	34	9	13	15	10	16

*Percent of methylation on each CpG site was evaluated by bisulfite sequencing. Position refers to that of the CpG dinucleotide relatively to transcription start site (Fig. 1), and N refers to number of sequenced clones.

**Figure 6:**
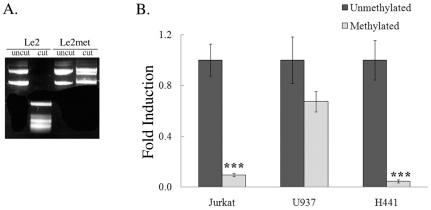
Methylation of CpG sites in the *vav*1 promoter impairs expression of the reporter gene in various cell lines. (A) Le2 plasmid, either un-treated or methylated by CpG methyltransferase (M.SssI), was incubated with HpaII and analyzed on a gel. The plasmid treated with M.SssI was not digested by HpaII, indicating that methylation was successful. (B) Unmethylated or methylated Le2 was transfected into Jurkat T cells, U937 myeloid cells and H441 lung cancer cells. The luciferase activity of these plasmids was measured 24 hr after transfection. Fold induction of luciferase activity was calculated relative to the activity in cells transfected with unmethylated Le2. Each point is the mean of three experiments. (***) indicates p<0.01, unpaired student T test.

To further explore the role of DNA methylation in *vav*1 regulation, we analyzed the effect of methylation of the *vav*1 promoter on transcription using methylated and unmethylated forms of the luciferase reporter gene Le2. To estimate the efficiency of the methyltransferase reaction, we digested the unmethylated and methylated plasmids with HpaII, a methyl-sensitive restriction enzyme (described in Materials and Methods). HpaII fails to digest the methylated plasmid but does digest the unmethylated plasmid (Fig. 6A). The unmethylated form of Le2 transfected into Jurkat, U937 and H441 cells led to expression of the reporter gene (Fig. 6B), similar to the results presented in Fig. 2A. In contrast, luciferase activity was more than 90% lower in Jurkat and H441 cells transfected with the methylated plasmid and about 50% lower in U937 cells. These results indicate that methylation of the *vav*1 promoter is important for its restricted tissue specific expression.

**Figure 7:**
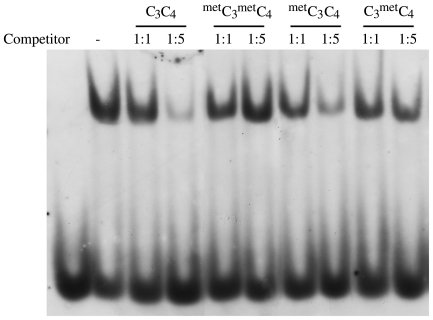
Methylation on CpG dinucleotides at putative transcription factor binding sites changes the affinity of protein complexes for the *vav*1 regulatory region. (A) EMSA was performed with Jurkat T cell nuclear extracts and lil3-4 labeled oligonucleotide. The probe was created by annealing complementary oligonucleotides lil79 and lil80 (Table 3).-3′). The following unlabeled competitors were added: unmethylated lil79-80 oligonucleotide (C_3_C_4_); oligo methylated on both CpG methylation sites (^met^C_3_^met^C_4_); oligo methylated only on CpG_3_ (^met^C_3_C_4_), or only on CpG_4_ (C_3_^met^C_4_). Competitor oligonucleotide was added in an amount equal to the labeled oligo (1∶1) or in 5 molar excess (1∶5).

The CpG content in *vav*1 regulatory sequences is not high enough to create CpG islands. We hypothesized that methylation of individual CpG sites may interfere with transcription by a mechanism different from that exerted by CpG islands. The transcription factor binding site for ETF/Sp1 contains a putative CpG methylation site (CpG_4_) (Fig. 1), which may affect the interaction between DNA and DNA-binding proteins. To resolve this issue, we performed EMSA experiments in the presence of methylated and unmethylated unlabeled competitor (Fig. 7). A digoxigenin-labeled double-stranded DNA probe encompassing CpG_3_ (−156 bp relative to TSS) and CpG_4_ (−148 bp) were used. The probe was incubated in the presence of nuclear extract from Jurkat T cells and one of the following unlabelled competitor oligonucleotides: unmethylated at CpG_3_ and CpG_4_ positions (C_3_C_4_), methylated at CpG_3_ and CpG_4_ (^met^C_3_^met^C_4_), methylated only at CpG_3_ (^met^C_3_C_4_) or only at CpG_4_ (C_3_^met^C_4_). ^met^C_3_^met^C_4_ and C_3_^met^C_4_ had no effect on binding to the unmethylated probe, whereas ^met^C_3_C_4_ reduced binding similarly to the non-methylated competitor C_3_C_4_ (Fig. 7). This result demonstrates that methylation on the CpG_4_ dinucleotide interferes with protein binding to the *vav*1 promoter, but methylation at CpG_3_ does not play an important role in this type of regulation.

## Discussion

To investigate the mechanisms underlying tissue-specific and cancer-related transcription of *vav1*, we used a reporter gene approach. We found that luciferase under the control of the *vav*1 promoter is expressed at a higher level in Jurkat T cells than in U937 monocytic cells (Fig. 2A). In H441 lung cancer cells, luciferase expression from the same plasmid was even higher than in Jurkat T cells (Fig. 2A). These results are consistent with the physiological expression of *vav1*, which is particularly high in lymphocytes and megakaryocytes [33]. Similarly, transgenic mice expressing hCD4 under the *vav*1 promoter show that the highest level of the gene expression is found in lymphocytes, eosinophils and megakaryocytes, while monocytes and neutrophils have an intermediate level of *vav*1 expression and erythroid cells have the lowest level [11]. These data validate our reporter approach to deciphering the regulation of *vav*1 expression.

Sequence analysis identified several consensus transcription factor sites in the *vav1* promoter, including sites for Sp1, P300 and YY1, which are expressed ubiquitously [34], and for the tissue-specific factors c-Myb and PU.1 [27], [35], [36] (Fig. 1). In our analysis, mutations at the PU.1 binding site caused dramatic decreases in reporter gene expression in U937 cells (constructs Le15 and Le17, Fig. 2C), consistent with the previous report that PU.1 is critical for *vav*1 expression in U937 cells [15]. Mutations at this site also dramatically decreased luciferase expression in Jurkat T and H441 cell lines. PU.1 expression is restricted to the myeloid cell lineage and is not expressed in Jurkat T or H441 cells, so it is unclear which transcription factor binds to this sequence and enables *vav*1 transcription in these cell types. PU.1 belongs to the ETS family of transcription factors, which have highly similar DNA-binding domains yet have diverse functions and activities physiologically and in oncogenesis [37]. Sokalski *et al.* demonstrated that the function of PU.1 in B cell differentiation is complemented by the related ETS transcription factor Spi-B, which binds to the same DNA consensus sequence [38], [39]. It is reasonable to suggest that other members of the ETS family bind to the consensus sequence in the *vav1* promoter in lymphoid Jurkat T cells and H441 lung cancer cells.

While mutations at the PU.1 binding site had a severe effect on transcription from the *vav*1 promoter in all cell lines tested, the cells responded differentially to mutagenesis at the E2F/NF1-e/c-Myb or ETF/Sp1 binding sites (Fig. 2B, C). While mutation in the E2F/NF1-e/c-Myb binding site (Le7 and 12) led to a marked reduction in the expression of the luciferase reporter in hematopoietic cell lines, their effect on luciferase expression in lung cancer cells was minor. In contrast, a point mutation in the ETF/Sp1 binding site (Le19) affected expression in hematopoietic cells to a lesser extent than in H441 lung cancer cells. These results imply that some of the regulatory mechanisms important for *vav*1 transcription are distinct between different hematopoietic cell lineages, as well as between hematopoietic cells and lung cancer cells. Other tissue-specific regulatory mechanisms may affect sites that are not included in the *vav1* promoter sequences in the reporter construct we used here.

We have identified five protein complexes that bind to the core promoter region of the *vav*1 gene in cells of different histological origins, revealing the complex organization of the regulatory network of this gene (Fig. 3). Only the heaviest protein complexes are affected by the mutations that we introduced into the promoter region (Fig. 2B, Fig. 3). These results raise the possibility that complexes 1, 2 and 3 are non-specific or that they bind to parts of the oligonucleotide that are not affected by our mutations and do not interact with the complexes represented by bands 4 and 5. Despite the fact that the nucleotides that have been changed in the oligonucleotides Le7, 12, 13, 15 and 17 define two different putative transcription factor binding sites, all of these mutations lead to disappearance of the protein complex represented by band 5 in the EMSA experiments (Fig. 3). This finding may indicate that the factors that bind to these sites physically interact with each other to create a high order protein complex that regulates *vav*1 expression. In Jurkat T cells, deletion of the E2F/NF1-e/c-Myb binding site also weakened binding of complex 4, whereas deletion of the TCFα/PU.1/ELF1 site did not (Fig. 3, left, oligonucleotides Le13 and 17). It may indicate that the complex represented by band 5 includes the one represented by band 4. It is conceivable that the protein complexes that associate with the mutated sequences are slightly different in lung cancer cells and Jurkat T cells. This could be because different proteins make up the binding complexes in these cell types or because factors in the complexes are differentially modified in these cell types in a way that regulates binding to DNA or to other proteins in the complex.

Our experiments indicate that c-*myb* could be one of the transcription factors that contribute to the expression of Vav1 (Figs. 2, 3, 4, 5). First, a mutation in c-Myb binding site impedes expression driven by *vav*1 promoter in Jurkat T cells, U937 and H441 cells (Fig. 2). Second, a mutation introduced in c-Myb binding site affects protein complex formation (Fig. 4). Third, we found differential expression of *c-myb* RNA in cell lines of different histological origins: it was present at very high levels in Jurkat T cells, somewhat lower levels in H441 lung cancer cells, and not at all in the H460 lung cancer cell line (Fig. 5A), suggesting that expression of c-Myb and Vav1 may be correlated in these cells. c-Myb is essential for hematopoiesis [30], [31], [40]. In addition, it has been implicated in progenitor cell maintenance and is required for proper cellular differentiation in the hematopoietic system, neuronal cells, skin cells, and colonic crypts [40]–[43]. c-Myb is highly expressed in immature hematopoietic cells and its expression is down-regulated upon differentiation. High *c-myb* expression has been associated with oncogenic activity and poor prognosis in several human cancers, including T-cell leukemia, acute myelogenous leukemia, colorectal tumors, breast cancer, and most recently, adenoid cystic carcinomas [35], [44], [45]. Our results clearly show an association between the presence of c-Myb and *vav*1 expression since over-expression of c-Myb in Vav1-negative H460 lung cancer cells along with the *vav*1 reporter gene induced expression of luciferase (Fig. 5B), while depletion of *c-myb* expression in Vav1-positive H441 lung cancer cells led to a marked reduction in *vav*1 mRNA expression (Fig. 5C). c-Myb expression is associated with the control of other genes known to be linked to cancer. For example, osteopontin (OPN) is a secreted extracellular matrix protein that has been linked to tumor progression and metastasis in a variety of cancers. Increased OPN expression is associated with the clinical stage, portending a poor prognosis. Inhibition of *c-myb* by siRNA decreased the transcriptional activity of the OPN promoter, reduced the expression of OPN, and compromised the migration and invasion capacity of Hepatocellular carcinoma (HCC) cells [46]. Vav1 was also shown to be associated with the expression of OPN [36], [46]. Like OPN in HCC, over-expression of Vav1 protein in PDAs [22] and lung cancers [47] is associated with poor prognosis. In addition, it is associated with increased migration of the cancer cells. Collectively, these results raise the possibility that c-Myb regulates the expression of Vav1 in cancer, thus playing a central regulator of cells invasive properties in some cancer types.

Ubiquitously active promoters tend to have high CG content and are regulated by few transcription factors, while tissue-specific promoters tend to have low CG content and are regulated by many different proteins [48]. The CpG island is defined as a sequence of at least 200 to 500 base pairs with CpG content above 55% in which observed to expected ratio is above 0.65. This ratio is calculated using the formula: (number of CpGs×number of bp)/(number of Cs×number of Gs) (http://www.uscnorris.com/cpgislands/cpg.cgi). The CpG content in the *vav*1 5′ regulatory sequences presented in Figure 1 is relatively high, about 60%, but the observed to expected CpG ratio is rather low, only 0.32.

Tissue-specific hypomethylation is well correlated with gene expression profiles that underlie tissue phenotypes. Around these cell-type specific hypomethylated regions, binding motifs of particular transcription factors are remarkably enriched. A combination of tissue-specific promoter hypomethylation and selective binding of transcription factors is involved in targeting specific genes during terminal differentiation [49]. Our results indicate that the promoter of *vav*1 is totally unmethylated in lymphocytes where *vav*1 is normally expressed, whereas other tissues reveal various levels of methylation (Table 5). This finding, along with the high density of putative transcription factors binding sites in the *vav*1 promoter region, suggests that this promoter has characteristics consistent with other tissue-specific genes.

Our reporter gene studies show that methylation of the *vav1* promoter affects transcriptional activity (Fig. 6). Notably, transfection of a methylated *vav1* promoter- luciferase reporter plasmid into Jurkat T cells led to a decrease of more than 90% compared to activity in cells transfected with unmethylated plasmid. In U937 cells, the methylated plasmid produced about 50% less luciferase activity than the unmethylated plasmid. This result emphasizes the role of epigenetic regulation of the *vav*1 gene and suggests that regulation of gene expression in these closely-related cell lineages - lymphoid and myeloid - may differ at a number of levels including tissue-specific transcription factors such as PU.1 [50] and sensitivity to DNA methylation. This research indicated that the predominant mechanism of *vav*1 expression regulation is the presence of activating transcription factors rather than gene repressing mechanisms.

Epigenetic changes are common in most, if not all, human malignancies. They seem to occur early in cancer development; consistent with the notion that epigenetic deregulation precedes and promotes malignant processes. In tumor cells, deregulation of DNA methylation is found in two forms: the overall loss of 5-methyl-cytosine (global hypomethylation) and gene promoter-associated (CpG island-specific) hypermethylation [51]. Notably, most research on the role of DNA methylation in cancer has focused on promoters with CpG islands as a regulatory unit. Fernandez-Zapico *et al.* showed that no methylation of the *vav*1 gene was detected in the cell lines that express Vav1 or in DNA from primary human pancreatic tumors but *vav1* promoter methylation was detected in Panc1 cells that do not express *vav*1 endogenously. Panc1 cells do express Vav1 following transfection, indicating that the *vav*1 gene is not appropriately methylated in Vav1-expressing cell lines and pancreatic tumor specimens. This study also showed that treatment of pancreatic cells that do not express Vav1 with DNA demethylation agents lead to Vav1 expression, suggesting that ectopic expression of Vav1 in primary pancreatic cancer is the result of an epigenetic modification of the *vav*1 gene regulatory sequences. This study proposes that methylation in the *vav*1 promoter is the main mechanism of gene silencing in the pancreatic cells [22]. These findings are in accordance with our results showing little or no expression from the methylated *vav1* promoter- luciferase reporter construct in Vav1-expressing Jurkat T cells and H441 lung cancer cells (Fig. 6).

To evaluate which of several CpG sites affects *vav*1 transcription, we performed an EMSA experiment with an oligonucleotide that spans the CpG_3_ and CpG_4_ sites. Our results (Fig. 7) show that methylation at the CpG_4_ but not at CpG_3_ is critical for interaction between proteins and DNA. CpG_4_ is located within a putative binding site for the transcription factors ETF and Sp1, but there are no consensus sequences predicted in the CpG_3_ location. Katryniok *et al.* reported that recruitment of Sp1 to its binding site in the human 5-lipoxygenase gene promoter is prevented by methylation [32]. However, in an earlier work, Iguchi-Ariga and Schaffner did not find an effect of CpG methylation on Sp1 binding in the cAMP promoter [52]. Sensitivity of a DNA-binding protein to DNA methylation can be changed by covalent modifications of the protein or by cofactors that compose the transcriptional complex. Based on these data, Sp1 is a good candidate for regulating *vav*1 transcription.

Collectively, our experiments show that both tissue-specific positive transcription factors and epigenetic mechanisms play important roles in the regulation of *vav*1 expression.
